# A cross-sectional comparison of disability and quality of life in euthymic patients with bipolar affective or recurrent depressive disorder with and without comorbid chronic medical illness

**DOI:** 10.4103/0019-5545.39755

**Published:** 2008

**Authors:** Hema Tharoor, Ashutosh Chauhan, Podila Satya Venkata Narasimha Sharma

**Affiliations:** Department of Psychiatry, Kasturba Medical College, Manipal - 576 104, India; 1 Department of Behavior and Mental Health, Sahyadiri Hospitals, Pune, India

**Keywords:** Bipolar disorder, disability, quality of life

## Abstract

**Background:**

There are major health care implications of quality of life (QOL) and disability in long-standing disorders such as bipolar affective disorder (BAD) and recurrent depressive disorder (RDD).

**Objectives:**

To compare the inter-episode QOL and disability in patients with the diagnosis of BAD or RDD in remission with and without comorbid chronic medical illness.

**Materials and Methods:**

Cross-sectional assessments of the four groups were carried out. Euthymic bipolar or RDD subjects with chronic comorbid medical illnesses were included in the study. QOL assessment was carried out using the World Health Organization (WHO)-QOL - Bref Kannada version. Disability was assessed using the Schedule for Assessment of Psychiatric Disability (SAPD), which is an Indian modification of the WHO Disability Assessment Schedule-II.

**Results:**

Eighty patients were enrolled into the study (20 patients in each group). The mean disability scores in the BAD group was significantly more in ‘social role’ (*P* = 0.038), and in the RDD group it was more in ‘home atmosphere’ (*P* = 0.001) in the two groups (*n* = 40) with chronic comorbid medical illness. In the other group without comorbid chronic medical illness (*n* = 40), the BAD group had significantly more disability in ‘overall behavior’ (*P* = 0.002) and ‘social role’ (*P* = 0.001), and the RDD group had significantly more disability in ‘assets and/or liabilities’ (*P* = 0.004) and ‘home atmosphere’ (*P* = 0.001). The QOL measures did not differ significantly between the two disorders.

**Conclusions:**

The presence of chronic comorbid medical illness did not cause a difference in the QOL between the two groups in periods of euthymia. However, disability measures differed significantly between the groups.

The concepts of ‘disability’ and ‘quality of life (QOL)’ offer a broad perspective for assessing the needs and outcomes of chronic mental patients. The assessments of disability in mood disorders in euthymia with chronic comorbid medical illnesses have been the focus of clinical research in general medicine and mental health specialty practices.[1] QOL research in bipolar disorders has evolved over the years and is fast gaining popularity as an important morbidity index along with disability.[2,3] Recent developments in medical outcome research have made it possible to compare the impact of widely disparate medical and psychiatric conditions on overall patient functioning and well-being. This facilitates health care resource allocation to areas of greatest needs and potential benefits.[4] Therefore, cross-sectional and longitudinal studies using ‘disability’ and ‘QOL’ measures are needed to project the extent of dysfunction in mood disorders with and without chronic medical comorbidity.

## MATERIALS AND METHODS

### Subjects

The sample comprised 40 subjects, aged 18 years and above, equally composed of two main disorders - bipolar affective disorder group (BAD) and recurrent depressive disorder group (RDD) in remission with and without comorbid chronic medical illness. A purposive sampling method was employed to ensure equal distribution of twenty subjects across four different subgroups. The four subgroups were as follows: BAD in remission, RDD in remission, BAD in remission with comorbid chronic medical illness, and RDD in remission with comorbid chronic medical illness. The period of remission required to enter the study was taken as 2 months. The patient groups were recruited from the outpatient department of psychiatry at Kasturba Hospital, Manipal, India. The target population consisted of patients fulfilling ICD-10 Diagnostic Criteria for Research (DCR)[5] guidelines of BAD or RDD in remission. The consultant psychiatrists in the department using the ICD-10 DCR delineated the diagnosis and duration of remission of 2 months. Patients with and without comorbid chronic medical illnesses were recruited in the study. Other psychiatric disorders, including substance use existing as a comorbid diagnosis, were excluded.

### Assessment

Data were collected on the basis of a single cross-sectional interview of the subjects who fulfilled the inclusion/exclusion criteria and provided written informed consent. The diagnoses were ascertained by face-to-face clinical interview of the patients and their informants by the psychiatrists using the ICD-DCR criteria. All consenting adults who fulfilled the inclusion criteria were first administered the socio-demographic proforma and the life chart by the investigator. The life chart was adapted from the National Institute of Mental Health life chart method.[6] Medical comorbidity was ascertained from the patient or accompanying relative and verified with available hospital records. The patient's medical diagnosis and treatment were being conducted in the same hospital as the study and maintained in the medical records division case files. The ill period and well period were derived by life chart review and from case records of the patients. MRSI (mean ratio of symptomatic ill period/illness duration) scores were calculated in order to see if the proportion of ill period to total illness duration since onset reflects the actual difference in duration of illness across patients. Each subject's ratio of symptomatic ill period/illness duration since the onset of illness was computed and multiplied by 100. The resulting values for all patients in the group were summated and mean derived for each group was designated as MRSI.

Disability was assessed by using the Schedule for Assessment of Psychiatric Disability (SAPD),[7] which is an Indian modification of the WHO Disability Assessment Schedule-II (DAS-II). The schedule was rated and disability scores were obtained. The period of assessment was taken as the last 1 month. The SAPD items were grouped into four (or five) main areas of disability, including personal, social role, occupational and global disability. Ratings were done on a six-point scale (0 to 5). It has been previously used in patients with psychiatric disorders as well as diabetes mellitus. Adequate reliability and ability to discriminate between different patient groups has been shown. This instrument has four sections. At the end of the schedule, a global judgement about the level of disability of the patient should be made.

Section 1 deals with overall behavior, and section 2 deals with social role performance. The ratings for these two sections are from 0 (no dysfunction) to 5 (maximum dysfunction). Therefore, the mean scores are likely to range from 0 to 5.

Section 3 (patient in the hospital) has not been used in the study.

Section 4 is modifying factors, which includes items designed to describe specific assets (five items)/specific liabilities (three items) as well as salient features of his/her home environment (three items). These are rated as 0 (no) and 1 (yes), or 8 (impossible to make a judgement) and 9 (not known/not enquired). In the present study, the scores of these two components have been summated excluding ratings of 8 and 9; hence, the mean scores may range from 0 to 8.

Home atmosphere and outside support components (three items) were rated on a 0-5 scale (no dysfunction to maximum dysfunction).

Finally, the global evaluation subscale is rated from 0 to 5 (no dysfunction to maximum dysfunction).

The QOL assessment was made with WHO-QOL[7] - Bref Kannada version. This scale was chosen because it is a generic scale developed simultaneously in 15 field centers around the world (India was one of the participating countries). It is a subjective assessment for adults with a reading age of 8 years and above, and can be completed with interviewer assistance. This 26-item self-administered scale measures four domains of QOL. They are physical health (item nos. 3, 4, 10, 15-18), psychological health (item nos. 5-7, 11, 19, 26), environment (item nos. 8, 9, 12-14, 23-25) and social relationships (item nos. 20-22). Item numbers 1 (QOL) and 2 (QOL) reflect a general factor named ‘general well-being’, which is not considered a specific domain. The items are scored from 1 to 5 with total scores ranging from 26 to 130, higher scores indicating better QOL in each domain and in total score. The psychometric properties of WHO-QOL Bref have been found to be comparable with those of the full version of WHO-QOL 100. High correlation of domain scores (0.89 or above) for the two scales has been obtained using a four-domain structure. This scale has shown good discriminant validity, content validity, internal consistency and test-retest reliability.

### Statistical analysis

**spss** software package (version 10, SPSS Inc., Chicago, USA) was used to analyze the data. Descriptive statistics were used for all variables. Group comparison for the categorical variables was done using chi-squared test and Fischer's exact test of probability. Group comparisons for continuous variables were done using independent *t*-test.

## RESULTS

### Sociodemographic characteristics

Table 1 represents the distribution of the socio-demographic variables across the BAD and RDD subjects without comorbid chronic medical illness. They did not differ significantly on variables of age, gender, marital status, residential status and family history. There was a statistically significant difference between the groups on (a) income levels (*P* = 0.013) with persons in RDD reporting higher income levels and (b) family type (*P* = 0.047) with persons in the RDD group belonging to extended families.

**Table 1:** Distribution of socio-demographic variables in all the BAD and RDD subjects without chronic comorbid medical illness

Variables	BAD *n* = 20 (%)	RDD *n* = 20 (%)	Statistics
Mean age (SD)	35.75 (12.2)	38.50 (5.53)	*t* = 0.917, df = 38, *P* = 0.361
Gender			
Male	11 (55)	9 (45)	λ^2^= 0.400; *P* = 0.527, df = 1
Female	9 (45)	11 (55)	
Marital status			
Single	5 (25)	5 (25)	λ^2^ = 0; *P* = 1, df = 1
Married	15 (75)	15 (75)	
Education			
Illiterate	2 (10)	4 (20)	λ^2^ = 5.97; *P* = 0.113, df = 3
Primary	13 (65)	16 (80)	
Secondary	4 (20)		
Graduate	1 (5)		
Occupation			
Unemployed	7 (35)	1 (5)	λ^2^ = 2.487; *P* = 0.478, df = 3
Homemaker	12 (60)	5 (25)	
Laborers	1 (5)	14 (70)	
Professional			
Residence			
Urban	1 (5)		λ^2^ = 0; *P* = 1, df = 3
Rural	19 (95)	20 (100)	
Income			
<1000			λ^2^ = 8.667; *P* = 0.013*, df = 2
1001-3000	9 (45)	1 (5)	
3001-5000	6 (30)	9 (45)	
>5001	5 (25)	10 (50)	
Family			
Nuclear	10 (50)	4 (20)	λ^2^ = 3.956; *P* = 0.047*, df = 1
Extended	10 (50)	16 (80)	
Family history			
Present	8 (40)	5 (25)	λ^2^ = 1.02; *P* = 0.311, df = 1
Absent	12 (60)	15 (75)	

BAD - bipolar affective disorder, RDD - recurrent depressive disorder, *n* = 40;

* *P* < 0.05

Table 2 reflects the subgroup with chronic comorbid medical illness. There was a statistically significant difference in (a) marital status (*P* = 0.037) with more persons in the RDD group being married, (b) occupation (*P* = 0.001) with all RDD subjects being laborers, (c) family history of mental illness (*P* = 0.006) being more frequent in the BAD group and (d) income (*P* = 0.021) being higher for the RDD group than the BAD group. However, both the groups did not differ significantly on the variables of age, gender, education, place of residence and family type.

**Table 2:** Distribution of socio-demographic variables in all the BAD and RDD subjects with chronic comorbid medical illness

Variables	BAD *n* = 20 (%)	RDD *n* = 20 (%)	Statistics
Mean age (SD)	40.6 (7.62)	41 (4.21)	*t* = 0.18, df = 38, *P* = 0.853
Gender			
Male	9 (45)	11 (55)	λ^2^ = 0.400; *P* = 0.527, df = 1
Female	11 (55)	9 (45)	
Marital status			
Single	6 (30)	1 (5)	λ^2^ = 4.3; *P* = 0.037*, df = 1
Married	14 (70)	19 (95)	
Education			
illiterate	2 (10)	4 (20)	λ^2^ = 7.23; *P* = 0.065, df = 3
Primary	12 (60)	16 (80)	
Secondary	5 (25)		
Graduate	1 (5)		
Occupation			
Unemployed	4 (20)	20 (100)	λ^2^ = 19.25; *P* = 0.001*, df = 3
Homemaker	7 (35)		
Laborer	7 (35)		
Professional	2 (10)		
Residence			
Urban	1 (5)		λ^2^ = 0; *P* = 1, df = 3
Rural	19 (95)	20 (100)	
Income			
<1000	1 (5)		λ^2^ = 9.73; *P* = 0.021*, df = 3
1001-3000	3 (15)	6 (30)	
3001-5000	11 (55)	14 (70)	
>5001	5 (25)		
Family			
Nuclear	8 (40)	7 (35)	λ^2^ = 0.107; *P* = 0.744, df = 1
Extended	12 (60)	13 (65)	
Family history			
Present	10 (50)	2 (10)	λ^2^ = 7.61; *P* = 0.006*, df = 1
Absent	10 (50)	18 (90)	

BAD - bipolar affective disorder, RDD - recurrent depressive disorder, *n =*40;

* *P* < 0.05

### Illness variables

The overall trend in the illness variables seen in Table 3 represents patients with BAD or RDD without chronic comorbid medical illness.

**Table 3:** Distribution of illness variables in all the BAD and RDD subjects without comorbid chronic medical illness

Variables	BAD (*n* = 20), Mean SD	RDD (*n* = 20), Mean SD	Statistics
Age of illness onset (years)	23.5 (7.5)	25 (2.7)	*t* = 0.81, *P* = 0.39, df = 38
Total illness duration (months)	126 (67.02)	145.3 (53.1)	*t* = 1, *P* = 0.319, df = 38
Total symptomatic ill period (months)	8.8 (3.4)	10.3 (2.69)	*t* = 1.5, *P* = 0.13, df = 38
Well period (months)	117.4 (65.56)	134.5 (52.7)	*t* = 0.9, *P* = 0.36, df = 38
Number of episodes	4.6 (1.2)	3.7 (0.47)	*t* = 3.2, *P* = 0.003*, df = 38
MRSI	8.5 (4)	7.9 (3.9)	*t* = 0.5, *P* = 0.61, df = 38

BAD - bipolar affective disorder, RDD - recurrent depressive disorder, MRSI - mean ratio of symptomatic ill period/illness duration, *n* = 40

The groups differed significantly (*P* = 0.001) in the number of episodes - BAD group with chronic comorbid medical illness [Table 4] having more number of episodes. The two groups did not differ in other illness variables.

**Table 4:** Distribution of illness variables in all the BAD and RDD subjects with comorbid chronic medical illness

Variables	BAD (*n* = 20), Mean SD	RDD (*n* = 20), Mean SD	Statistics
Age of illness onset (years)	24.6 (4.4)	27.2 (2.9)	*t* = 2.01, *P* = 0.051, df = 38
Total illness duration (months)	149.4 (54.2)	148.1 (64)	*t* = 0.069, *P* = 0.94, df = 38
Total symptomatic ill period (months)	9.9 (4.1)	8.9 (2.7)	*t* = 0.91, *P* = 0.36, df = 38
Well period (months)	139.5 (52.6)	139.2 (63.9)	*t* = 0.01, *P* = 0.36, df = 38
Number of episodes	5.8 (2.5)	3.5 (0.51)	*t* = 3.81, *P* = 0.001*, df = 38
MRSI	7.3 (3)	7.3 (3.8)	*t* = 0.001, *P* = 0.94, df = 38

BAD - bipolar affective disorder, RDD - recurrent depressive disorder, MRSI - mean ratio of symptomatic ill period/illness duration, *n =*40;

* *P* < 0.05

There is a statistically significant difference in the duration of medical comorbidity. The RDD group had medical illness for less than 10 years, and the BAD group had medical illness for longer than 10 years [Table 5]. The chronic comorbid medical illnesses seen were diabetes, hypertension and bronchial asthma. There were no significant differences between the two groups on the distribution of these illnesses [Table 6].

**Table 5:** Comparison of duration of comorbid chronic medical illnesses in persons with BAD/RDD

Duration of comorbidity	BAD (*n* = 20) %	ROD (*n* = 20) %	Statistics
<10 years	9 (45)		λ^2^ = 12.53; *P* = 0.001*, df = 1
>10 years	11 (55)	20 (100)	

BAD - bipolar affective disorder, RDD - recurrent depressive disorder, *n* = 40

**Table 6:** Distribution of diagnoses of comorbid medical illness in persons with BAD/RDD

Variables	BAD (*n* = 20) %	ROD (*n* = 20) %	Statistics
Diabetes	6 (30)	9 (45)	λ^2^ = 3.6; *P* = 0.463, df = 3
Hypertension	7 (35)	7 (35)	
Bronchial asthma	4 (20)	4 (20)	
Diabetes and hypertension	3 (15)		

BAD - bipolar affective disorder, RDD - recurrent depressive disorder, *n* = 40;

*P < 0.05

### QOL and disability variables

Table 7 illustrates the mean comparison scores of QOL and DAS variables between BAD and RDD subjects without chronic comorbid medical illness. There was no significant difference in any of the QOL variables between the groups. This was reflected in the findings on QOL variables between BAD and RDD with medical comorbidity. Among the DAS variables, the BAD group without comorbid chronic medical illness had significantly higher mean scores in ‘overall behavior’ (*P* = 0.002) and ‘social role’ (*P* = 0.001), suggesting higher disability than RDD in these areas. In comparison, the RDD group had significantly higher mean scores on ‘home atmosphere’ (*P* = 0.001) and in ‘assets and/or liabilities’ (*P* = 0.004), suggesting higher disability than the BAD group. Contrastingly, the BAD group with chronic comorbid medical illness [Table 8] had significantly higher mean score only on social role (*P* = 0.038). The RDD group with chronic medical illness continued to have significant higher disability in ‘home atmosphere’ (*P* = 0.001). There were no significant differences among the groups with reference to the ‘global evaluation’ score.

**Table 7:** Distribution of the mean quality of life (QOL) and Disability Assessment Schedule (DAS) scores in all the BAD and RDD subjects without comorbid chronic medical illness

Variables	BAD (*n* = 20), Mean SD	RDD (*n* = 20), Mean SD	Statistics
QOL - QOL1	3.3 (0.47)	3.2 (0.4)	*t* = 72, df = 38, *P* = 0.49
QOL2	3.6 (0.5)	3.5 (0.5)	*t* = 0.62, df = 38, *P* = 0.54
Physical domain	81.8 (3.3)	81.6 (3.5)	*t* = 0.19, df = 38, *P* = 0.85
Psychological domain	74.2 (3.03)	74.4 (2.01)	*t* = 0.25, df = 38, *P* = 0.81
Social domain	34.2 (2.9)	35.6 (3.2)	*t* = 1.5, df = 38, *P* = 0.15
Environmental domain	88.8 (6.8)	91.8 (8.4)	*t* = 1.2, df = 38, *P* = 0.22
DAS-overall behavior	4.7 (3.2)	3.1 (2.08)	*t* = 3.4, df = 38, *P* = 0.002*
Social role	3.4 (2.5)	2.9 (1.00)	*t* = 4.1, df = 38, *P* = 0.001*
Assets and/or liabilities	5.4 (1.6)	5.6 (1.3)	*t* = 3, df = 38, *P* = 0.004*
Home atmosphere	3.6 (0.5)	4.6 (0.6)	*t* = 5.6, df = 38, *P* = 0.001*
Social support	3 (1)	2.8 (1)	*t* = 0.62, df = 38, *P* = 0.54
Global evaluation score	1.9 (0.4)	1.9 (0.22)	*t* = 1, df = 38, *P* = 0.3

BAD - bipolar affective disorder, ROD - recurrent depressive disorder, *n* = 40;

* *P* < 0.05

**Table 8:** Distribution of the mean quality of life (QOL) and Disability Assessment Schedule (DAS) scores in all the BAD and RDD subjects with comorbid chronic medical illness

Variables	BAD (*n* = 20), Mean SD	RDD (*n* = 20), Mean SD	Statistics
QOL - QOL1	3.3 (0.47)	3.4 (0.5)	*t* = 0.682, df = 38, *P* = 0.34
QOL2	3.4 (0.5)	3.3 (0.47)	*t* = 0.614, df = 38, *P* = 0.52
Physical domain	80.6 (2.68)	81.2 (2.62)	*t* = 0.714, df = 38, *P* = 0.479
Psychological domain	74 (2.05)	73.4 (2.34)	*t* = 0.860, df = 38, *P* = 0.395
Social domain	35.2 (3.03)	35.2 (2.78)	*t* = 1.08, df = 38, *P* = 0.284
Environmental domain	90 (7.94)	94 (8.46)	*t* = 1.54, df = 38, *P* = 0.132
DAS-overall behavior	4.7 (2.79)	3.6 (2.08)	*t* = 1.41, df = 38, *P* = 0.166
Social role	4.3 (2.05)	3.2 (1.00)	*t* = 2.15, df = 38, *P* = 0.038*
Assets and/or liabilities	3.6 (1.23)	4.3 (1.34)	*t* = 1.71, df = 38, *P* = 0.094
Home atmosphere	3.45 (0.51)	5 (0.794)	*t* = 733, df = 38, *P* = 0.001*
Social support	1.4 (0.82)	2.6 (0.94)	*t* = 0.717, df = 38, *P* = 0.478
Global evaluation score	1.9 (0.31)	1.9 (0.31)	*t* = 0.000, df = 38, *P* = 1.00

BAD - bipolar affective disorder, RDD - recurrent depressive disorder, *n* = 40;

* *P* < 0.05

## DISCUSSION

The study centre caters to a predominantly rural/suburban catchment area. So, a majority of persons in both the groups were from rural area and did not show difference in various socio-demographic variables studied [Table 1] except for education and income. Coryell and associates[8] examined bipolar and unipolar patients with comparison groups and observed no differences in the socioeconomic variables in both bipolar and unipolar disorder patients. In a review of studies[9,10] on bipolar disorders, a strong association between bipolar and higher socioeconomic status has been reported. This finding could not be replicated in our study. A majority of persons in the BAD group was laborers, and this could explain their lower socioeconomic status. A significantly higher positive family history of mental illness in persons with BAD is in keeping with the earlier research.[11] In the medically ill subgroup, the socio-demographic variables did not differ between the two groups of patients [Table 2]. These findings are similar to the large-scale epidemiological studies,[1,12] which have examined functional disability in patients with depression and comorbid chronic medical illness. In the medical outcome study,[13] QOL was measured using the Short Form-36 (SF-36). SF-36 has eight subscales, which may be collapsed into two domain scores reflecting the physical and mental components of QOL. In this study, it was found that, when depressive symptoms and diabetes co-occurred, the effects of diabetes and depressive symptoms on functional disability were associated with approximately double the reduction in social functioning as compared to either condition alone. The duration of chronic comorbid medical illness is significantly more in the BAD group [Tables 5 and 6]. There is no significant difference in the distribution of medical diagnoses between the two groups. It is not clear whether an earlier onset of chronic medical morbidity in BAD in this study may be a secular trend or more likely artefactual due to sampling limitation. Robb,[14] using the Illness Intrusiveness Scale (IIRS), reported that illness intrusiveness is compromised in bipolar disorders in periods of euthymia. Illness intrusiveness has been investigated in relation to QOL. He reported that illness intrusiveness experienced by 87 euthymic patients with bipolar disorder was similar to that in subjects with multiple sclerosis and greater than that in subjects with end-stage renal disease and rheumatoid arthritis.

Illness-related variables [Tables 3 and 4] show no significant differences in the two disorders, except for the higher number of episodes in persons with BAD. It is worth noting that chronic comorbid medical illness factor did not seem to affect the course of illness differentially in BAD and RDD. This finding appears to be robust and suggests that the effect of medical illness on the illness variables studied is similar in BAD and RDD. The overall similarity of QOL in the two groups, if not artefactual or attributable to sampling limitations, could suggest that the two illnesses by themselves do not differentially affect QOL. Chronic comorbid medical illness did not alter the QOL variables in the two disorders.

The DAS variables studied show that persons with BAD without medical comorbidity had significantly higher disability in ‘overall behavior’ and ‘social roles’. This finding is consistent with earlier studies[3,11] on disability in bipolar disorders. There is no significant dysfunction in ‘overall behavior’ in the BAD group with medical comorbidity. This suggests that comorbid chronic medical illness reduces the overall behavior dysfunction in BAD or more likely increases it in RDD, thus obliterating the difference. Persons with pure RDD or RDD with chronic comorbid medial illness had significantly higher dysfunction in ‘home atmosphere’. The significant ‘assets and/or liabilities’ dysfunction in RDD is not evident when comorbid medical illness exists. Therefore, it is likely that chronic comorbid medical illness obliterates the differences in the two groups.

Thus, medical illnesses may have a role in increasing disability but less likely to have a significant impact on QOL in the two disorders during euthymia. However, the study had some limitations. A larger sample frame, assessment of the severity and treatment of medical comorbidity on disability and QOL could further validate potential differences in the two groups.
